# Transcriptomic signature for drug-induced steatosis

**DOI:** 10.17179/excli2015-758

**Published:** 2015-12-21

**Authors:** Regina Stöber

**Affiliations:** 1 Leibniz Research Centre for Working Environment and Human Factors at TU Dortmund (IfADo), Ardeystrasse 67, 44139 Dortmund, Germany

## ⁯

Non-alcoholic fatty liver disease (NAFLD) affects almost one third of adults in European countries. A well-known factor of influence is overnutrition and insulin resistance (Farrell and Larter, 2006[7]). In contrast, much less attention is given to drug induced steatosis (Chen et al., 2014[4]). It is crucial that steatosis inducing compounds are detected and excluded early in drug development (Hewitt et al., 2007[16]; Hengstler et al., 2000[15]; Vitins et al., 2014[20]; Fisher et al., 2008[8]; Rogiers et al., 1997[19]).

Recently, Marta Benet and colleagues from Barcelona and Valenica in Spain have published a transcriptomic signature to identify compounds with an increased risk of inducing steatosis (Benet et al., 2014[2]). Numerous mechanisms can cause steatosis. Many steatosis-inducing drugs impair fatty acid beta-oxidation (FAO) by inhibiting involved enzymes or by blocking the transport of fatty acids into mitochondria. 

Moreover, compromising the mitochondrial respiratory chain or mitochondrial potential may indirectly cause steatosis (Benet al., 2014[2]; Donato and Gomez-Lechon, 2012[5]; Fromenty and Pessayre, 1995[9]). One possibility to study the potential to induce steatosis is to quantify the levels of neutral lipids, e.g. by BODIPY or oil red O staining. However, these techniques have been reported to be relatively insensitive (Benet et al., 2014[2]). In their recent publication Benet and colleagues (2014[2]) have established a common signature of three transcription factors: FOXA1, HEX and SREBP1C. Totally, 25 well-characterized compounds were studied in HepG2 cells. Using the aforementioned transcription factors as biomarkers, 92 % of the test compounds were correctly classified (Benet et al., 2014[2]). Currently much effort is invested in research on hepatotoxic mechanisms (Onami et al., 2014[18]; Campos et al., 2014[3]; An, 2012[1]; Fang et al., 2012[6]; Monteiro et al., 2013[17]) and *in vitro *systems to predict hepatotoxicity (Godoy et al., 2015[14], 2013[13], 2009[12]; Zellmer et al., 2010[21]; Ghallab, 2014[11][10]). 

In this context the identification of the steatogenic FOXA1/HEX/SREBP1C signature by Benet and colleagues (2014[2]) represents an important progress and should be included into predictive studies in future.
